# Ultra‐Processed Foods and Neuropsychiatric Disorders: A Scientometric Study

**DOI:** 10.1155/bmri/8104780

**Published:** 2025-11-30

**Authors:** Fran Espinoza-Carhuancho, Gabriel Barriga-Yauri, Julia Medina, Lucia Quispe-Tasayco, Arnaldo Munive-Degregori, Katia Medina-Calderon, Frank Mayta-Tovalino

**Affiliations:** ^1^ Grupo de Bibliometría, Evaluación de evidencia y Revisiones Sistemáticas (BEERS), Human Medicine Career, Universidad Cientifica del Sur, Lima, Peru, ucsur.edu.pe; ^2^ Research, Entrepreneurship and Innovation Unit, Universidad Nacional Federico Villarreal, Lima, Peru, unfv.edu.pe; ^3^ Academic Department, Universidad Nacional Mayor de San Marcos, Lima, Peru, unmsm.edu.pe; ^4^ Vicerrectorado de Investigación, Universidad San Ignacio de Loyola, Lima, Peru, usil.edu.pe

**Keywords:** neuropsychiatric disorders, scientometrics, ultra-processed foods

## Abstract

**Purpose:**

The purpose of this study is to analyze the academic production of ultra‐processed foods and their relationship with neuropsychiatric disorders, assessing trends, collaboration patterns, and emerging thematic areas between 2019 and 2024.

**Methods:**

The RAMIBS guidelines for scientometric studies were followed. The search was performed in Scopus using terms related to ultra‐processed foods and neuropsychiatric disorders. Studies published between January 2019 and December 2024 were included, excluding letters to the editor and conference proceedings. Data were analyzed with SciVal and R Studio, using Bibliometrix to explore metrics such as Scholarly Output, SNIP 2023, CiteScore 2023, h‐index, and international collaboration. Bradford’s Law was applied to identify key journals.

**Results:**

We identified 53 documents from 50 sources, with an annual growth of 24.57%. The average age of the documents was 2.98 years, with an average of 10.49 citations per publication. Brazil and the United States led in production with six articles each, while the international collaboration reached 18.87%. The most outstanding journals were “Nutrients” (SNIP 1.31, CiteScore 9.2) and “Preventive Medicine” (SNIP 1.37, CiteScore 7.7). Keyword analysis revealed a focus on the relationship between diet, obesity, and mental health. According to Bradford’s Law, “Nutrients” led as the most relevant source.

**Conclusions:**

The bibliometric data show a sustained growth in research on ultra‐processed foods and neuropsychiatric disorders, with Brazil and the United States as the main contributors. The journal Nutrients played a key role as a source of dissemination. Although high thematic diversity was evident, international collaboration was limited, reflecting opportunities to strengthen global networks.

## 1. Introduction

Ultra‐processed foods (UPFs) are ready‐to‐eat industrial products made primarily from refined ingredients and additives, with little or no whole food content [1]. Although initially defined as products created with ingredients unique to industrial processing using advanced technology [2], the NOVA Food Category System, introduced in 2009, is the most widely recognized method for categorizing foods according to their level of processing [3].

The NOVA system classifies highly processed food as “ultra‐processed” (UPF) and defines them as industrial products made mostly from food‐derived substances, nonculinary ingredients, and additives to provide the final product with convenience, taste, appeal, and low cost [3–6]. Recent studies have shown that UPFs are correlated with increased obesity, diabetes, hypertension, and mortality risk in observational studies [7], and higher caloric intake and weight gain in randomized trials [8]. It arises, therefore, from how few countries would put in place polices that regulate UPF consumption on purpose [9–11].

Neuropsychiatric disorders (NPDs) are a broad concept that refers to medical conditions encompassing mental health problems related to alterations in the nervous system, which implies that the behavior of individuals is affected. These disorders can reduce the life expectancy of individuals in various ways and pose significant health risks [12]. In 2019, approximately 971 million people worldwide suffered from mental disorders [13]. This number increased markedly because of the recent COVID‐19 pandemic [14].

In the last decade, there has been increasing interest in the link between the consumption of UPFs and the onset of various NPDs, along with aggressive and antisocial behaviors [15]. NPDs include depression, dementia, psychosis, and bipolar disorder, which are aligned with other regimes of unhealthy lifestyles that include smoking, alcohol consumption, poor diet, and physical inactivity. These behaviors can result in the onset of obesity, and it has been shown that the prevalence of obesity among patients with schizophrenia is higher than that in the general population [16].

There is a growing body of research on this topic; therefore, we chose to conduct a study with a scientometric approach. Scientometrics is a methodology that analyzes and quantifies scientific production through text mining, providing indexes on thematic areas, authors and institutions. Moreover, it is key to evaluate scientific activities, develop policies, establish collaborations, and predict future trends, with an increasing focus on the evaluation of scientific journals [17, 18].

Thus, the aim of this research was to evaluate the evolution, emerging patterns, and collaborative networks of research on UPF and NPDs.

## 2. Methods

The Reporting and Measurement of Items for Bibliometric or Scientometric Studies in Health Sciences (RAMIBS) [19] was used to report the findings of this scientometric study.

### 2.1. Study Design

This observational, descriptive study with a scientometric approach analyzed the academic production of UPF and their relationship with NPDs between 2019 and 2024.

### 2.2. Search Strategy

The search was carried out on January 24, 2025 in the Scopus database, using the following search formula: TITLE‐ABS (“ultra‐processed foods” OR “processed foods” OR “junk food” OR “fast food” OR “convenience food” OR “prepackaged food” OR “snack food” OR “unhealthy food”) AND TITLE‐ABS (“neuropsychiatric disorders” OR “mental health conditions” OR “brain disorders” OR “psychiatric disorders” OR “cognitive disorders” OR “psychological disorders” OR “behavioral disorders” OR “mental illness” OR “psychopathological conditions”) AND PUBYEAR >2018 AND PUBYEAR <2025.

### 2.3. Selection Criteria

We included studies published between January 2019 and December 2024 that addressed the relationship between UPFs and NPDs. Articles had to be available in the Scopus database and written in English. All methodological designs were accepted, except letters to the editor and conference proceedings, which were excluded.

### 2.4. Procedure in SciVal and Bibliometrix

The selection and extraction of data in SciVal began by identifying relevant documents using the search formula previously established in Scopus. After obtaining the results, the established inclusion and exclusion criteria were applied. In SciVal, international collaborations, publication impact, and citation metrics were analyzed. The data were then exported to R Studio for analysis with Bibliometrix. In R Studio, a detailed analysis of keywords, the distribution of publications according to Bradford’s and Lotka’s laws, and visualizations were generated to facilitate the interpretation of the data.

### 2.5. Data Analysis

Data analysis was conducted using SciVal and R Studio to explore research on UPFs and NPDs. Metrics such as Scholarly Output, SNIP 2023, Citations per Publication, CiteScore 2023, as well as indicators related to affiliations, countries or regions, h‐index, Citation Count and Views Count were analyzed. Bradford’s Law was used to identify the most relevant journals in the field. In addition, total scientific productivity was examined, highlighting collaboration between countries, regions, and institutions, as well as the impact of this research through the analysis of citations and views. Coauthorship networks were also constructed, and citation patterns were detected to identify key players and emerging trends. All analysis was performed using SciVal and R Studio 4.3.2.

## 3. Results

Between 2019 and 2024, research on UPF and NPDs showed an annual growth of 24.5%. Fifty‐three papers from 50 sources were analyzed, with an average of 10.4 citations per paper and a total of 3723 references. The authors used 233 keywords and 260 researchers participated, with an average of 4.9 coauthors per paper and 18.8% international coauthorships. Most of the papers were articles (35), followed by reviews (11), book chapters (3), conference papers (3), and a book (Table 1).

**Table 1:** Main characteristics.

**Description**	**Results**
Timespan	2019:2024
Sources	50
Documents	53
Annual growth %	24.5
Document average age	2.9
Average citations per doc	10.4
References	3723
Author’s keywords	233
AUTHORS	
Authors	260
Authors of single‐authored docs	2
Single‐authored docs	2
Coauthors per doc	4.91
International coauthorships %	18.8
Article	35
Book	1
Book chapter	3
Conference paper	3
Review	11

**Figure 1:**
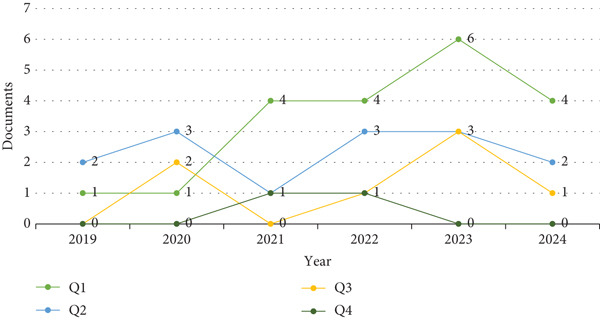
Impact of scientific publications by quartile.

In total, 43 papers were published, with a notable increase in high‐quality publications (Q1) from one in 2019 to six in 2023. Publications in Q2 were also relevant, with 14 papers. Although the number of publications in Q3 and Q4 was lower, with seven and two papers, respectively, the overall growth reflects a growing interest in this field of study (Figure 1).

Several journals have contributed notably, for example, “*Nutrients*” published three papers with a SNIP 2023 of 1.31, 2.7 citations per publication and a CiteScore 2023 of 9.2. “*Preventive Medicine*” had two papers with a SNIP of 1.37, nine citations per publication and a CiteScore of 7.7. “*Advances in Nutrition*” stood out with one article, a SNIP of 2.31, 22 citations per publication and a CiteScore of 17.4. Other journals such as “*Antioxidants*” and “*Appetite*” also showed a notable impact on this field (Table 2).

**Table 2:** Top 10 most productive sources.

**Scopus source**	**Scholarly output**	**SNIP** ^**a**^ **2023**	**Citations per publication**	**CiteScore 2023**
Nutrients	3	1.31	2.7	9.2
Preventive Medicine	2	1.37	9	7.7
Advances in Nutrition	1	2.31	22	17.4
American Journal of Lifestyle Medicine	1	0.73	0	4.1
Annals of General Psychiatry	1	1.62	19	6.6
Antioxidants	1	1.38	22	10.6
Appetite	1	1.39	13	9.1
BMC Geriatrics	1	1.32	0	5.7
BMC Psychiatry	1	1.37	7	5.9
BMC Public Health	1	1.39	1	6.5

^a^ ^a^SNIP: source normalized impact per paper.

Several authors have been noted for their contributions. Matthew H. Hobbs of the University of Canterbury in New Zealand had a scholarly output of two papers, with 79 views and an h‐index of 19 and 20 citations. Liana B. Basal of Alliant International University in the United States published one paper, with 10 views, an h‐index of 11, and 0 citations. Daniele Cristina Aguiar of Universidade Federal de Minas Gerais in Brazil also contributed one paper, obtaining 15 views, an h‐index of 30 and one citation. Other notable authors include Nourah Alfayez of Alfaisal University in Saudi Arabia and Camille Amadieu of Université catholicus de Louvain in Belgium, who also made important contributions to this study (Table 3).

**Table 3:** Top 10 most productive authors.

**Author**	**Affiliation**	**Country**	**Scholarly output**	**Views count**	**h-index**	**Citation count**
Hobbs, Matthew H.	University of Canterbury	New Zealand	2	79	19	20
Abascal, Liana B.	Alliant International University	United States	1	10	11	0
Aguiar, Daniele Cristina	Universidade Federal de Minas Gerais	Brazil	1	15	30	1
Akin, Sumeyye	University of Health Sciences	Turkey	1	20	0	0
Al‐Bayati, Mohammad B. Abdulrazzaq	University of Health Sciences	Turkey	1	20	0	0
Alfayez, Nourah	Alfaisal University	Saudi Arabia	1	76	3	12
Alves, Yan Mathias	Universidade de São Paulo	Brazil	1	3	7	1
Amadieu, Camille	Université catholique de Louvain	Belgium	1	27	14	15
Amoretti S, Silvia	Vall d’Hebron Research Institute	Spain	1	23	24	10
Araújo, Juliana Soares Tenório De	Universidade de São Paulo	Brazil	1	3	1	1

**Figure 2:**
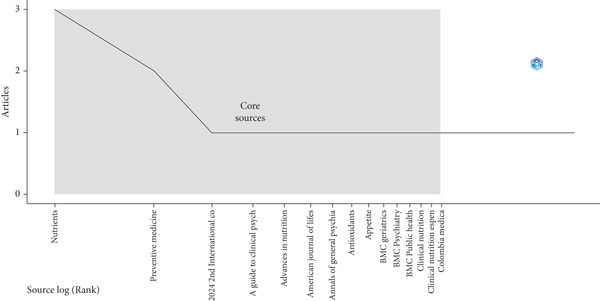
Core sources by Bradford’s Law.

According to Bradford’s law, in Zone 1, the journal “*Nutrients*” led with three publications, followed by “*Preventive Medicine*” with two publications. Other sources in this zone included conferences and clinical guidelines. In Zone 2, journals such as “*Frontiers in Neuroscience*” and “*International Journal of Eating Disorders*” were found, each with one publication. Finally, in Zone 3, journals such as “*Metabolic Brain Disease*” and “*Social Science and Medicine*” were included, each with one publication. This distribution reflects the diversity of sources and the breadth of academic interest in the subject (Figure 2).

**Figure 3:**
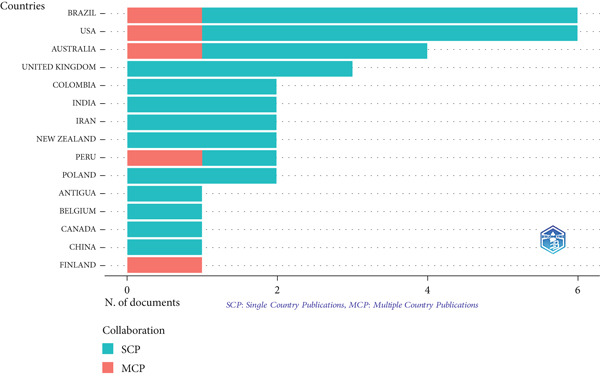
Corresponding author’s countries.

Brazil and the United States lead with six articles each, representing 11.3% of the total, with five national collaborative articles (SCP) and one international collaborative article (MCP), equivalent to 16.7%. Australia follows with four articles (7.5%), three SCP, and one CCM (25%). Other notable countries include the UK, Colombia, India, Iran, New Zealand, Peru, and Poland, each with two articles, with Peru’s international collaboration standing out with 50% MCP (Figure 3).

**Figure 4:**
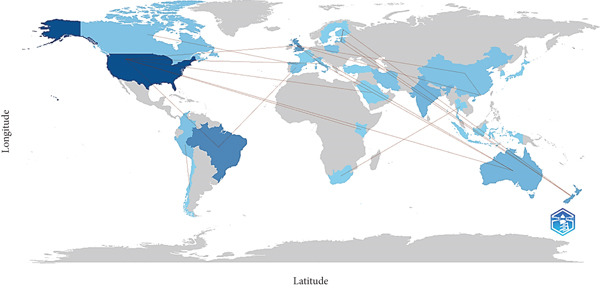
Country collaboration map.

The map of cross‐country collaboration in research on UPFs and NPDs shows diverse international connections. Australia collaborated with Italy, Brazil with Spain and the United States, and China with Hong Kong. Estonia had collaborations with Finland and France with Canada. Latvia collaborated with both Estonia and Finland. New Zealand established collaborations with Estonia, Finland, and Latvia. Peru collaborated with Chile and South Africa with Thailand. The United Kingdom collaborated with Australia, China, Hong Kong, and Italy. The United States collaborated with Australia, Italy, Kenya, Saudi Arabia, and the United Kingdom (Figure 4).

**Figure 5:**
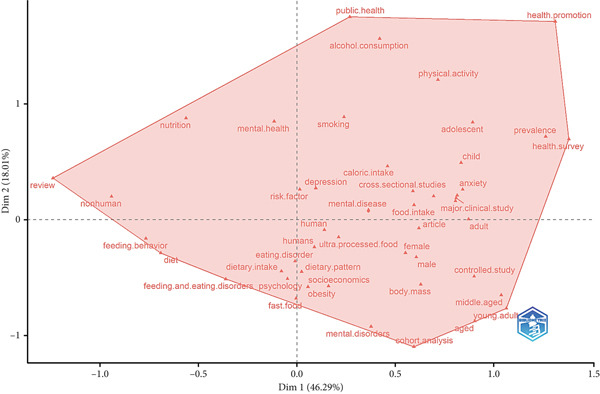
Conceptual structure map.

Factor analysis of words by cluster in the research on UPFs and NPDs revealed key terms grouped into a single cluster. Words such as “human”, “male”, “female”, “adult”, “mental. Health”, “depression”, “diet”, “obesity”, among others, reflect the focus areas and emerging patterns in research in this field (Figure 5).

**Figure 6:**
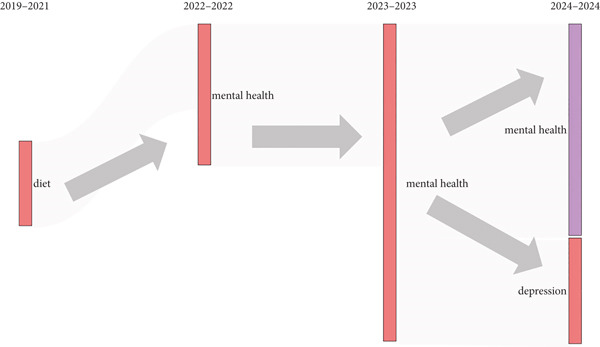
Thematic evolution.

The thematic progression indicates a significant shift in scientific focus across time. From 2019 to 2021, research began with attention being paid to “diet,” but with 2022, attention very much shifted to mental health. Not only did this theme stay rather evenly present but also demonstrated a heightened emphasis in 2023 and 2024. In fact, we see the theme of “depression” was introduced in 2024 as a sub‐branch of the theme of “mental health,” indicating that attention was deepening on the original theme of mental health with more specificity. The pattern shows an overall thematic movement from general lifestyle‐risk factors to more discrete psychological challenges, which reflects wider societal and clinical issues (Figure 6).

**Figure 7:**
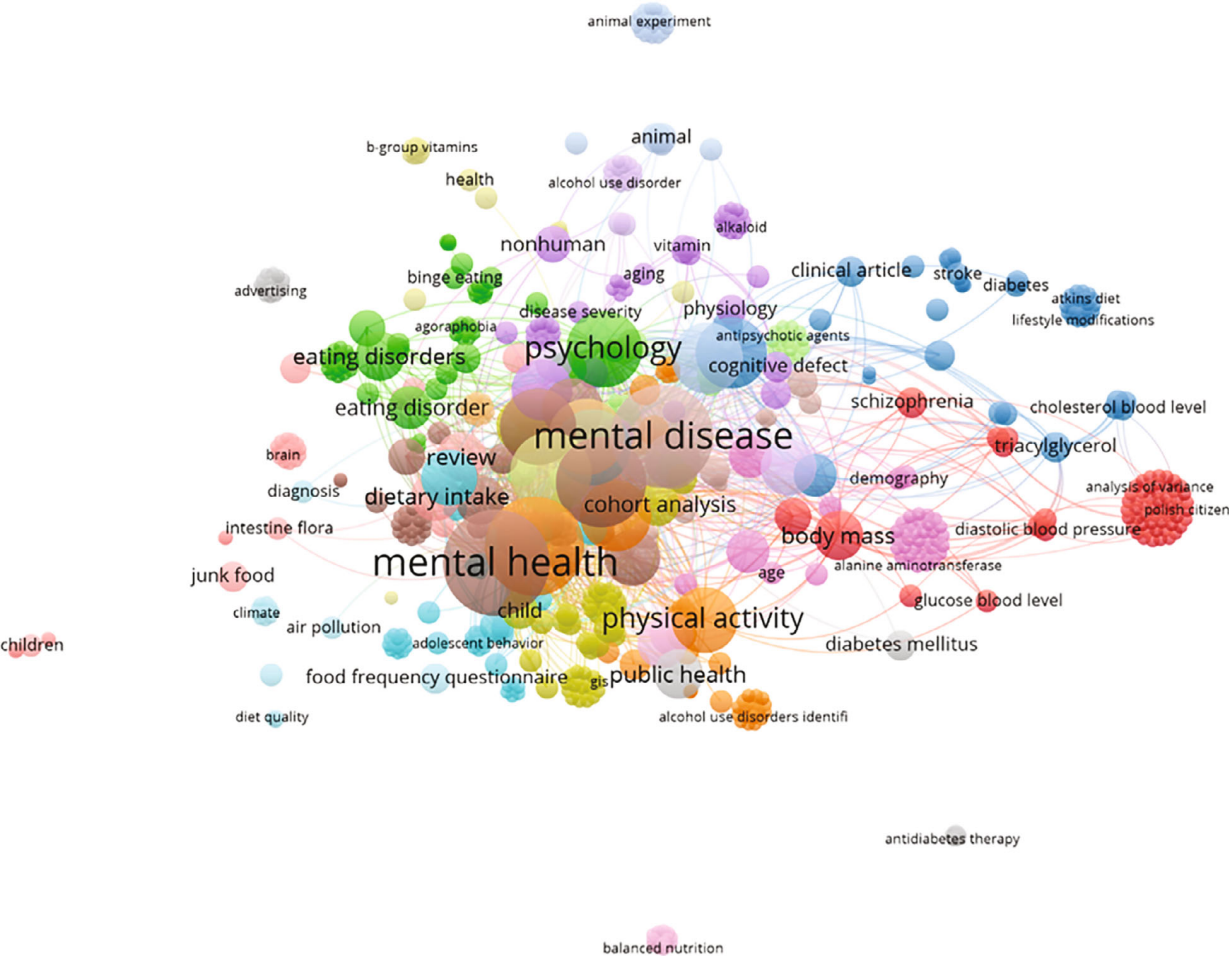
Co‐occurrence by keyword.

The map of co‐occurrence has three primary clusters associated with studies of UPFs alongside NPDs. The first relates to “Mental disease” alongside “Mental Health” with terms like “eating disorder,” “junk food”, “ultra‐processed foods,” indicating an interest in food quality as one factor related to health risks. The next cluster includes terms related to “psychology,” and the third cluster is connected to “Physical activity” and “Public Health,” which speak directly to the apparent increased interest in understanding the pathophysiological processes in the consumption of ultra‐processed food. In summary, the clusters imply that the literature has connected UPFs and NPDs (Figure 7).

## 4. Discussion

A significant increase in the literature on UPF and NPDs has been observed. The studies analyzed indicate that the increase in fast food in recent decades, because of its great accessibility, has led many people to rely on these unhealthy food practices, harming their health [18, 19].

We opted to conduct a scientometric study because these studies facilitate the analysis of trends in publications related to a particular topic or institution [20]. The search was conducted in Scopus because of its comprehensive database, which offers tools for citation and author analysis, outperforming other databases and providing a more complete view of the academic landscape [21].

Lan et al. [22] identified key research areas and trends in fatty acids related to central nervous system diseases. Between 2017 and 2022, an increase in publications on fatty acids in the central nervous system was observed, reaching 435 articles, which accounted for 90.63% of the total number of studies analyzed. In contrast, our study, conducted between 2019 and 2024, showed an annual growth of 24.57%, with an average age of the papers of 2.98 years and an average of 10.49 citations per paper. In the same study, University College Cork, Ireland, had the most productive authors, such as John F. Cryan and Timothy G. Dinan. In comparison, our study identified Hobbs, Matthew H. from the University of Canterbury, New Zealand, as the most productive author, with two publications, 79 views, an h‐index of 19 and 20 citations. The journal *Nutrients* was the highest contributor in both studies, highlighting its relevance in this field [22].

Another study by Maspeke et al. mentioned that research on food‐related ohmic heating has shown an impressive annual growth rate of 11.09%. A total of 769 publications have been produced, with contributions from 1841 authors. Brazil stands out as the leading country in terms of research contributions. Sastry, S.K., has emerged as the most prolific author, while Teixeira, J.A., is noted for being the most collaborative. Review studies focusing on the effects of ohmic processes on the nutritional composition of fruits, vegetables, and grains have received the highest number of citations [23].

Other studies emphasize that globalization and modernization have increased sedentary lifestyles and the consumption of processed foods [24, 25]. Coinciding with our study, “*Nutrients*” and “*Appetite*” were the most important contributing journals, with key words like “obesity,” “ultra‐processed foods,” and “nutrition” [26].

It is crucial to consider the limitations of this study, such as the exclusive use of Scopus and the exclusion of relevant research from other databases such as PubMed and Web of Science. In addition, the analyzed period of 5 years limits the observation of broader trends. Despite these limitations, the study provides valuable conclusions for future research [27, 28], evaluating factors associated with stress and metabolism, enriching the analysis and contributing to a better understanding of the subject.

Another limitation was that while much of the current literature focuses on depression or general mental health, the thematic evolution reveals important gaps in the literature. For example, there are evidently important NPDs, such as schizophrenia, bipolar disorder, and dementia, that fall into this category, yet they have not been studied as much because of their importance in clinical practice, and potential links to diet. While diet‐related studies [29] are prevalent, current studies are primarily methodologically dominated by cross‐sectional studies and observational studies, and it was noted a lack of longitudinal studies, biomarker studies, and neuroimaging studies. This historical and present emphasis highlights a need for diet‐related research that is more thorough, and mechanistically informed to better elucidate the developmental pathways by which UPF consumption may contribute to NPDs through time.

## 5. Conclusion

The present scientometric analysis illustrates continued growth and diversification in research on UPFs and NPDs for the years 2019 to 2024. The thematic evolution highlights a shift from general dietary concerns to specific approaches in mental health, with a (thematic) focus on depression, which illustrates the increasingly complex and specialized nature of the field. An enhancement in international collaboration and interest across multiple disciplines has occurred, although methodological limitations are present, such as research approaches, longitudinal designs, and limited use of biomarkers and neuroimaging. Research is often consolidated in a few high‐impact journals, and conceptual clusters associate food quality with mental and public health, which reflects the advancement of the field and the areas of future need. Overall, the evidence suggests an emerging maturation of the field is taking place, which will enable research to consider more integrative studies hopefully examining and understanding the mechanisms and long‐term effects of diet on neuropsychiatric health.

## Ethics Statement

The authors have nothing to report.

## Disclosure

All authors read and approved of the final manuscript.

## Conflicts of Interest

The authors declare no conflicts of interest.

## Author Contributions

F.M.T. and F.E.C. searched for the database and analysis data. K.M.C., G.B.Y., F.M.T., F.E.C., J.M., L.Q.T., and A.M.D. wrote the main manuscript text and prepared the table. F.M.T. analyzed the data. F.M.T., F.E.C., designed, critically reviewed, and modified the manuscript.

## Funding

No funding was received for this manuscript.

## Data Availability

The data that support the findings of this study are available from the corresponding author upon reasonable request.
